# A Review and Meta-Analysis of Genotype by Environment Interaction in Commercial Shrimp Breeding

**DOI:** 10.3390/genes15091222

**Published:** 2024-09-18

**Authors:** Md. Mehedi Hasan, Peter C. Thomson, Herman W. Raadsma, Mehar S. Khatkar

**Affiliations:** 1 Sydney School of Veterinary Science, Faculty of Science, The University of Sydney, 425 Werombi Road, Camden, NSW 2570, Australia; md.m.hasan@sydney.edu.au (M.M.H.); peter.thomson@sydney.edu.au (P.C.T.); herman.raadsma@sydney.edu.au (H.W.R.); 2 Davies Livestock Research Centre, School of Animal and Veterinary Sciences, University of Adelaide, Roseworthy Campus, Roseworthy, SA 5371, Australia

**Keywords:** aquaculture, genetic correlation, genetic improvement, growth, survival

## Abstract

(1) Background: Genotype-by-environment interaction (G×E) can adversely impact genetic improvement programs. The presence of G×E is mainly measured as the genetic correlation between the same trait measured in different environments where departure from unity can be taken as presence of G×E. (2) Methods: To understand the extent of G×E in shrimp production, a review and meta-analysis was conducted using the results from 32 peer-reviewed studies. (3) Results: Of these, 22 G×E studies were conducted on Pacific white shrimp (*Litopenaeus vannamei*) with fewer studies reported in other shrimp species. The most frequently studied traits were growth and survival, with relatively few studies on traits of economic importance. The meta-analysis demonstrated a moderately high genetic correlation (*r*_g_ = 0.72 ± 0.05) for growth, indicating low to moderate levels of G×E with some re-ranking of breeding values across environments. However, substantial G×E was evident for survival where only a moderate genetic correlation (*r*_g_ = 0.58 ± 0.07) was observed for survival across different environments. A re-ranking of breeding values is likely for this trait and genetic improvement of shrimp for survival in one environment may not be effective in other environments. The results from ANOVA-based studies show that G×E accounted for 6.42 ± 1.05% and 7.13 ± 3.46% of the variation for growth and survival traits, respectively. (4) Conclusion: The significance of G×E necessitates tailored genetic improvement programs in commercial shrimp breeding. We discuss the scope and challenges of G×E for shrimp breeding programs, including opportunities of implementing G×E in genomic selection programs.

## 1. Introduction

**Figure 1:**
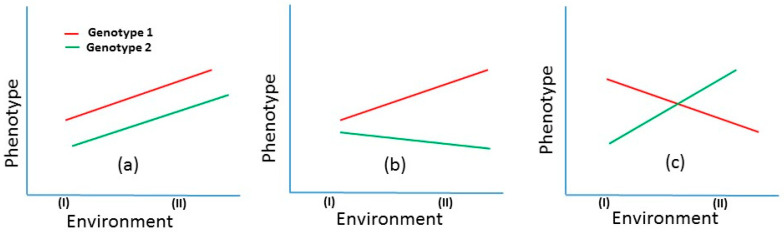
Illustration of genotype-by-environment interaction (G×E) using two hypothetical genotypes across two environments. (**a**) Absence of G×E: Both genotypes show higher expression in environment II, with genotype 1 (red) consistently outperforming genotype 2 (green). (**b**) Heterogeneity of genetic variance: The performance gap between genotypes widens in environment II compared with environment I, indicating a mild G×E. (**c**) Re-ranking of genotypes: A strong G×E is demonstrated, where genotype 1 performs better in environment I, while genotype 2 excels in environment II. This crossover interaction shows how each genotype is better adapted to a specific environment.

Selective breeding for livestock and poultry has been conducted over many generations and often with spectacular results; for example, selective breeding has resulted in a threefold increase in efficiency in beef production in cattle, and in poultry it is responsible for an 85% improvement of production performance [1,2]. With the exception of a few major aquaculture species (e.g., salmon, tilapia and carp), relatively little development has taken place in the implementation of genetic improvement programs in aquaculture [3]. In shrimp, most of the genetic improvement and genotype-by-environment (G×E) studies have focused on *Penaeus vannamei* (Pacific white shrimp) and *Penaeus monodon* (black tiger prawn) which contribute 87% of the world production of cultured shrimp [4]. Although over the last three decades, global shrimp aquaculture has increased by approximately 400%, the ever increasing demand will require significantly higher production [5,6]. Sustainable increases in the production of shrimp require the development of genetically improved and high-quality seed stock. In spite of the development of various breeding programs in several countries, the rate of genetic gain of shrimp is lower than the potential gains because of several factors thought to inhibit selection response (e.g., challenges in domestication, inadequate supply of genetically improved seed stocks, poor survival in the production environments) [7]. Moreover, even if improved stock is available, the performance of such stocks is often highly variable in different environments. Therefore, improved productivity of the target trait along with minimizing its variability across environments is desirable [8], as high variation around an optimum trait value can have a significant negative impact on production outcomes. There are limitations in preventing such variability due to dynamic climatic and environmental features that influence phenotypes. The phenotypic expression of genetically similar individuals can alter as a direct response to environmental circumstances [9]. This leads to genotype-by-environment interaction (G×E), which is defined as a different phenotypic expression of the same trait in different environments by genetically identical organisms (Figure 1) [10]. G×E has been a central issue in animal and plant breeding and can affect selection response in genetic improvement programs. In some cases, G×E can cause re-ranking of breeding candidates in different environments [11]. This means that superior breeding candidates in one particular environment (e.g., tropical condition) may not be the best in another environment (e.g., temperate condition), thus significantly reducing selection efficiency [12].

Aquatic animals are farmed under a wide range of production environments, such as different geographical locations, water conditions, and management systems. These diverse conditions can lead to significant G×E effects in aquaculture production. G×E has been reported to exist in most of the economically important aquaculture species, including Atlantic salmon [13], rainbow trout [14], Atlantic cod [15], Arctic charr [16], sea bass [17], gilthead sea bream [18], turbot [19], common sole [20], rohu carp [21], and Nile tilapia [22]. Recently, G×E has also been broadly reported for shrimp species (Table 1). There are significant differences in G×E across studies as well as between estimates within a species. For example, a review on G×E interaction across different aquaculture species has indicated that moderate re-ranking is present for growth and survival, with mean genetic correlations across environments of 0.72 and 0.54, respectively [23]. 

It is essential to understand the magnitude and pattern of G×E in shrimp aquaculture. Shrimp are typically raised in environments that experience extreme variation and where management practices vary within species and between farms; consequently, this can result in lack of uniformity in G×E estimates. Thus, while there is variation amongst published estimates, consensus estimates of G×E are not available to shrimp breeders and researchers. 

The primary objective of this study was to review and synthesize the findings of the published studies on G×E and its impact on shrimp breeding programs. By employing a novel meta-analysis methodology, we improved the precision of the consensus G×E estimates derived from the published research. Finally, we discuss several related aspects, including the prospects of utilizing G×E information in genomic selection of shrimp species.

## 2. Meta-Analysis Methodology

We compiled the published studies which investigated G×E for economically important traits in all of the major farmed shrimp species. Additionally, we conducted a meta-analysis and provide summary weighted estimates of G×E, as described in the following sections.

### 2.1. Study Selection

Prospective G×E studies of aquaculture species were selected using Google Scholar search engine with the ‘topic’ search terms ‘genotype,’ ‘environment,’ ‘interaction,’ and ‘aquaculture.’ In addition, we conducted forward citation searches of key G×E studies. We collated a final list of 32 articles based on reported G×E results. Alternate search engines, such as PubMed and other databases, were also used but yielded no additional relevant papers beyond the 32 articles already identified. These articles either provided genetic correlation estimates of traits measured in different environments or estimated the effects of G×E using ANOVA-based analyses in shrimp. For each of the selected G×E studies, we recorded publication date, species name, breeding design, pedigree, number of individual/family sampled, type of study (e.g., marker/pedigree based), traits, environment type and presence of G×E. The list of studies included are presented in Table 1. Studies which were rejected from further analyses either failed to report appropriate G×E effects for inclusion in the meta-analysis [54], or referred to environments deemed in appropriate for this study, i.e., “sex” effect [55].

### 2.2. Basic Study Information and Weighted Estimation of Genetic Correlation

Several studies have reported G×E estimates for economically important traits such as body color, body composition, and feeding efficiency. For our meta-analysis, we compiled various traits and environmental conditions, categorizing them into three broad trait groups (growth, survival, and other traits) and two environmental groups (habitat and stress types), respectively. The “other traits” group encompasses body color, body composition, and feeding efficiency (Table 2). Due to the limited number of studies available for each individual shrimp species, we pooled data from all shrimp species in our meta-analysis to increase statistical power and derive broader insights across the genus *Penaeus*. Evidence for G×E was based on two main approaches. Firstly, we compiled genetic correlation estimates of the traits measured in different environments (*r_g_*) as a measure of G×E and used these estimates in the first meta-analysis. Growth and survival were the two traits with the highest number of reports.

Secondly, the effects of G×E were estimated by the partitioning of the sum of squares (SS) from ANOVA-based analyses. The percentage of variation attributable to G×E was estimated as Family×Environment SS / Total SS × 100, with ‘family’ representing the genotype, for the environment in the expression of the target trait. Thereafter, the overall G×E was calculated by averaging overall estimates.

Among the selected studies on G×E interaction, a total of 28 studies reported 136 genetic correlation estimates for traits recorded in different environments. A portion of estimates, approximately 25% of the total data, did not include standard errors of genetic correlation estimates. For these estimates, a conservative approximate standard error value of the upper quartile of known standard error estimates was used and attributed to the estimate. Weighted genetic correlation estimates were calculated for all traits where two or more estimates were available including estimates across species. Where there was only one estimate or trait mean in any class, the value was recorded as it appeared in the original study. Previous studies have identified the inverse of the sampling variance, i.e., inverse of the standard error squared, as the optimal weighting method for a weighted meta-analysis [56,57], and this weighting method was applied here as described by Hasan, Tulloch [58]. As expected, for genetic correlation estimates, standard errors tend to be smaller near the boundaries, and skewed away from the boundaries, namely from −1 to +1 for correlations. Consequently, for genetic correlations (*r_g_*), the estimates were transformed using Fisher’s *z*-transformation, namely $z=\frac{1}{2}{log}_{e}\left(\frac{1+r_{g}}{1-r_{g}}\right),$ resulting in an approximately normal scale, and the meta-analysis was performed on this scale. Summary values from the meta-analysis were then back-transformed into correlations for presentation [58].

We assumed that there are *n* studies with estimates *r_i_* and corresponding standard errors se(*r_i_*), *i* = 1, 2, …, *n*. A *z*-transform of each estimate, *z_i_*, *i* = 1, 2, …, *n*, was obtained. A delta method approximate standard error of each *z_i_* was obtained as se(*z_i_*) = se(*r_i_*)/(1−$r_{i}^{2}$). The weighted mean of the *z_i_* was obtained, $\bar{z}=\sum_{i=1}^{n}w_{i}z_{i}/\sum_{i=1}^{n}w_{i}$, where *w_i_* = [se(*z_i_*)]^−2^, with the corresponding overall standard error $se\left(\bar{z}\right)={\left\{\sum_{i=1}^{n}{\left[se(z_{i})\right]}^{-2}\right\}}^{-1/2}$. Finally, these results were back-transformed to obtain the overall estimate of the correlation and corresponding delta method standard error, $\bar{r}=\frac{e^{2\bar{z}-1}}{e^{2\bar{z}+1}},\;se\left(\bar{r}\right)=se\left(\bar{z}\right)\times \frac{4e^{2z}}{{\left(e^{2\bar{z}}+1\right)}^{2}}.$

### 2.3. Factors Affecting G×E Detection

To evaluate the relationship of trait and environmental groups with G×E estimates (e.g., *r_g_* values), a linear mixed effect model was fitted using the lme4 package in R. In the linear mixed model, the outcome variable was *r_g_*, the fixed effects were either trait categories (namely growth, survival and other, where other includes the traits of body color, body composition and feeding efficiency) or environmental categories (e.g., habitat and stress type), and the random effect was study identification number. We calculated estimated marginal means, also known as least squares means, with the emmeans package in R [59]. We performed pairwise comparison of estimated marginal means between traits and environments. For this pairwise comparison, we classified traits into three and environment into two major categories (Table 2). All data were analyzed in the R statistical environment (version: 3.6.3) [60].

## 3. Meta-Analysis Results

Growth and survival were the most frequently reported traits across all of the studies. This might be due to these traits being directly related to the economic return in shrimp farming. Moreover, these traits are easier to record as compared with other traits. 

The most reported species was *P. vannamei*, consisting of 53% of all of the studies. The earliest G×E study on shrimp was conducted in 2002. There was no specific trend found in the number of studies published per year, although the highest number of studies was reported in 2020. By country, the highest number of the studies was found to have been conducted in China, followed by Australia (Table 1). All of the studies (*n* = 32) in which genotypic information is reported were derived from family-based breeding populations. 

**Figure 2:**
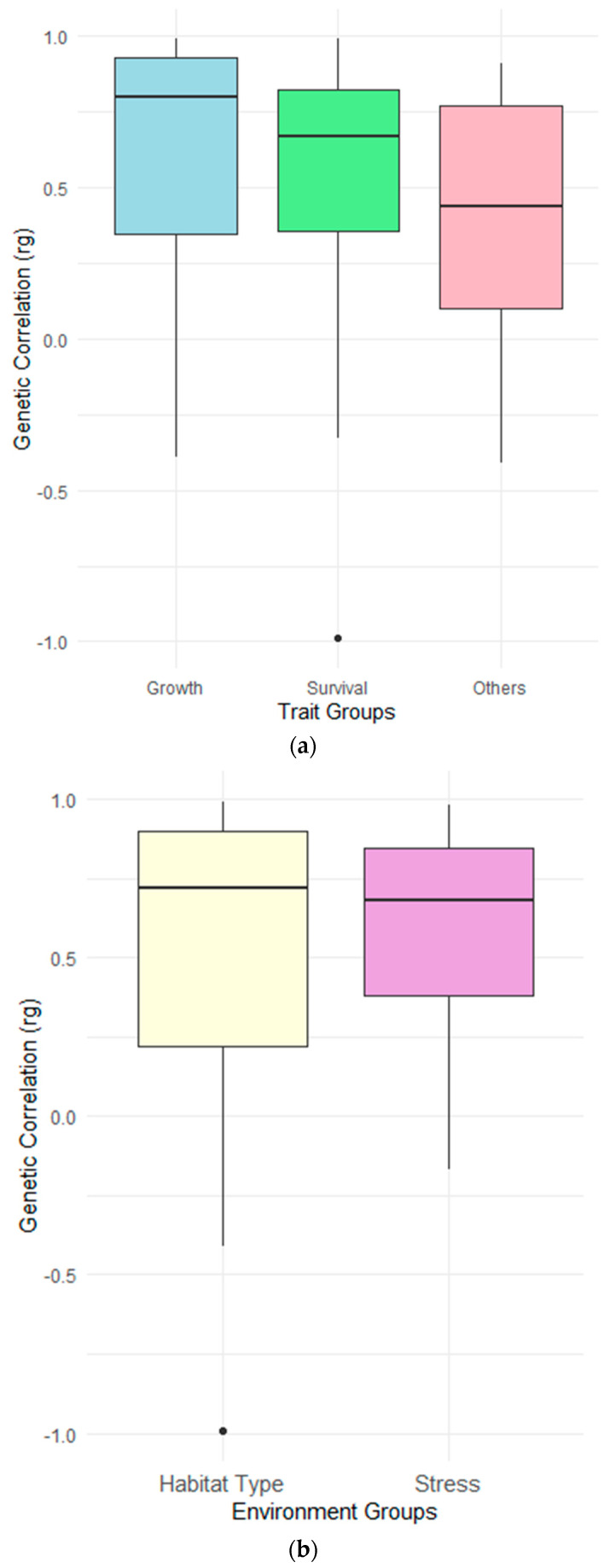
Distribution of *r_g_* across studies of G×E in shrimp species shown as boxplots based on (**a**) three trait groups (growth, survival and other) and (**b**) across two different environmental classification (habitat type and stress). Boxplots show median (central line), interquartile range (box), and range (whiskers, extending to 1.5 times the interquartile range) of *r_g_* values. Points beyond whiskers represent potential outliers. *r_g_* values closer to 1 suggest weaker G×E effects, while values closer to 0 or less indicate stronger G×E effects.

The weighted genetic correlation across different trait classes were 0.72 ± 0.05 for growth, 0.58 ± 0.07 for survival and 0.48 ± 0.27 for other traits (e.g., feeding efficiency, body color and body composition). The genetic correlation estimates were significantly different between trait groups (Table 3). The genetic correlation across environments were 0.65 ± 0.07 for habitat type (e.g., pond vs. tank) and 0.67 ± 0.08 for stress environment (e.g., pathogen presence vs. absence) (Figure 2). However, pairwise comparison revealed no significant difference between the two environment groups (Table 3).

**Figure 3:**
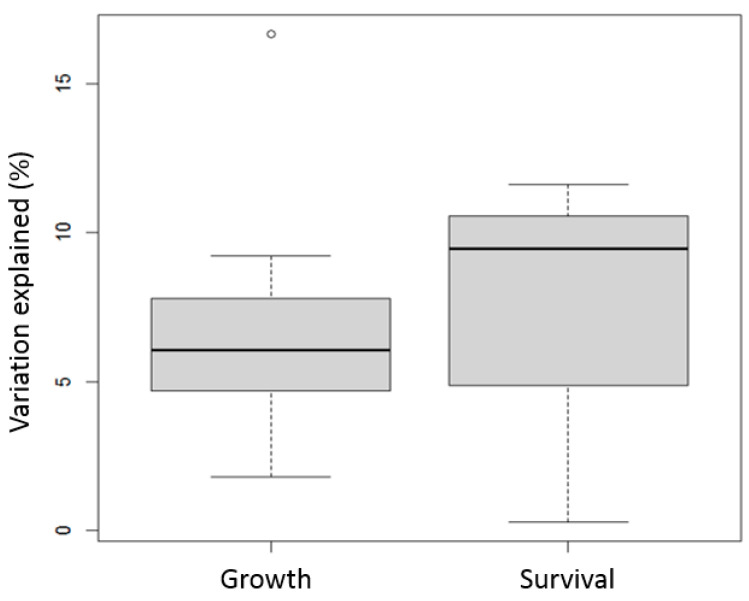
Boxplots of percent of phenotypic variation explained by G×E in ANOVA-based studies for growth and survival traits. Boxplots show median (central line), interquartile range (box), and range (whiskers, extending to 1.5 times the interquartile range) of % variation explained. Points beyond whiskers represent potential outliers.

The results from the meta-analysis of the studies (*n* = 4) on ANOVA SS reveal that habitat type and stress type had significant interactions with family (genotype) for the shrimp growth and survival traits (Table 4). The G×E explained 6.42 ± 1.05% of the variation in growth, and 7.13 ± 3.46% of the variation for the survival traits. A higher level of variability in G×E interaction was observed for survival traits (Figure 3).

## 4. Discussion and Implications for the Shrimp Industry

G×E has been reported for many traits of economic importance for a wide range of shrimp and aquaculture species. An overview of the data available so far suggests that the outcomes from these studies are highly variable, nevertheless, these meta-analyses have provided important baseline information for breeders to gain an overall understanding of G×E in shrimp aquaculture. G×E typically manifests in one of two ways: either as a re-ranking of genotypes (breeding values) across different environments or as a heterogeneity of genetic variances for the same trait measured in different environments. The former is more important than the latter, because re-ranking indicates that a single genotype is not optimal across all environments. Alternatively, when there is no re-ranking (*r_g_* = 1), selection in one particular environment leads to the same genetic gain across all environments. The aim of our meta-analysis was to assess the current state of G×E in shrimp breeding programs. We focused on G×E in shrimp as no such analysis has previously been conducted.

### 4.1. G×E in Growth Traits

Our meta-analysis revealed that the mean *r_g_* value for growth traits in shrimp is 0.72 ± 0.05. This high positive *r_g_* value implies that selection for improved growth performance in one environment is likely to lead to similar genetic responses in the same direction in other environments. The results indicate low to moderate overall G×E for growth traits in shrimp, implying that the extent of re-ranking might be minimal for these traits within shrimp species [34,41]. Similar to our findings of G×E for growth traits in shrimp, studies on other aquaculture species have shown high *r_g_* value and low G×E for growth traits (e.g., sea bass [61], sole [20], gilthead seabream [18], and Nile tilapia [62]) and is consistent with those presented in the review by Sae-Lim, Gjerde [23] for diverse aquaculture species. Despite the high average *r_g_* value for growth traits in shrimp species, a high degree of variability in *r_g_* values was observed (Figure 2). Variation in rearing environments on a farm showed a lower impact of G×E effect on growth traits in shrimp, whereas significantly greater variability in G×E was shown across diverse environments, from rearing to grow out. For example, Caballero-Zamora, Montaldo [38] reported negligible G×E for growth traits reared in different ponds (*r_g_* = 0.99 ± 0.03) for *P. vannamei.* Similarly, the presence of low G×E for growth trait was presented by Krishna, Gopikrishna [47] for *P. monodon* in ponds (*r_g_* = 0.78). Sui, Luan [39] reported a high genetic correlation (0.94 ± 0.06) for body weight traits in two farms for *P. vannamei.* Gitterle, Rye [29] reported low G×E for growth traits for *P. vannamei*, as the genetic correlation was close to unity, and it was consistent across the test environments. Argue, Arce [24] reported a weak G×E for growth traits between round and raceway tanks in *P. vannamei.* In contrast with these findings, Nguyen, Ninh [44] reported substantial G×E for growth traits (*r_g_* = −0.15 and 0.39) for *P. vannamei* reared in pond and tank environments. In this study, the authors have reported that ponds are a better environment in which to measure performance when compared with tanks. Jerry, Preston [53] reported sex-specific G×E for body weight trait across different ponds in *P. japonicas*. Argue, Arce [24] reported significant G×E for body weight traits between pond and a bio-secure aquaculture system. The interaction accounted for 1.81% of the variation. Pérez-Rostro, Racotta [27] reported the extent of G×E in *P. vannamei* reared in different locations and rearing conditions and found that there is an absence of G×E for harvest size traits and therefore a re-ranking of individual breeding values was not evident. Overall, findings from these studies suggest that, in most cases, G×E might be insignificant for growth traits when shrimp are farmed in different rearing environments. 

Wide variability was observed in G×E for growth traits in environments with different rearing density. Rearing density, which is considered a source of resource competition, is regarded as an important environmental factor leading to G×E interaction. Luan, Wang [37] reported low G×E for body weight traits for *P. vannamei* reared in two densities (*r_g_* = 0.71 ± 0.11). Similarly, Campos-Montes, Montaldo [34] have reported that there is no evidence of G×E for bodyweight in *P. vannamei* produced in different farming methods (e.g., semi-intensive, intensive and super-intensive). Tan, Luan [41] examined the impact of G×E on the growth of *P. vannamei* in two different densities and found high genetic correlations (0.94 to 0.98). These authors found slightly better production in this species in the low-density condition. In contrast, Ibarra and Famula [33] reported the presence of a substantial G×E effect on the body weight traits in *P. vannamei* reared in two different densities (*r_g_* = 0.56 ± 0.12). These authors have recommended that this species should be grown in higher densities as overall production was higher under such conditions. Castillo-Juárez, Casares [32] have reported the presence of G×E for body weight in *P. vannamei* reared in low and high density, although *r_g_* = 0.80 ± 0.08 to 0.86 ± 0.04 suggests the effects may have been small. Overall, these findings suggest G×E for density could impact the efficiency of selective breeding programs for the economically important traits of shrimp, if stocks produced from the breeding programs are cultivated across a wide range of densities. 

Environmental stress is another significant environmental impact factors studied in G×E investigations. Here environmental stress is usually defined by water temperature, salinity, and hypoxia. Growth traits showed stronger G×E effects when the rearing environment was stressful. Li, Luan [36] found significant G×E for growth traits for *P. vannamei* at different rearing temperatures. Coman, Crocos [51] have reported the presence of significant genotype-by-temperature interactions for growth and survival traits across a wide range of rearing temperature conditions. 

Growth traits are generally considered non-fitness traits and are less influenced by environmental variation [63,64]. For example, growth traits tend to have significantly higher additive genetic variation compared with survival traits [65]. However, G×E for growth traits is evident in shrimp when the rearing environment is stressful (e.g., temperature, salinity, ammonia tolerance etc.). This implies that re-ranking can be evident even for non-fitness traits (e.g., growth) when the environment is stressful and adversely affects survival. This finding in shrimp is relatively consistent with those of several other aquaculture species, including tilapia, salmon and rainbow trout, where significant G×E was observed for growth traits under highly stressful environments [23]. Therefore, it is likely that G×E effects may have a significant impact on the selection of growth traits in shrimp breeding programs when the environment is stressful and affects survival as described in the following section.

### 4.2. G×E in Survival Traits

Survival traits are the second most reported traits for G×E interaction studies in shrimp. In fact, it is the most important set of economic traits in shrimp aquaculture, with a direct impact on profitable shrimp farming, and thus warrants inclusion into the overall breeding objective. Our meta-analysis revealed a genetic correlation of *r_g_* = 0.58 ± 0.07 for survival traits, suggesting a strong G×E and noticeable re-ranking of breeding values for this trait across the different environments which impact differential survival. Moreover, we observed extreme variability in G×E for this trait across the range of −0.99 to 0.99 for *r_g_* values (Figure 2). This indicates that re-ranking for survival is of significant importance and it should be noted that a full re-ranking is possible for survival traits under some environments. In general, this suggests that this trait is strongly influenced by numerous environmental factors. Similar to our findings regarding shrimp species, relatively strong G×E effects for survival traits have been reported for other farmed aquaculture species here including Atlantic salmon and rainbow trout [66,67]. Considering individual studies, Lu, Luan [40] reported a medium strength *r*_g_ of survival traits in *P. vannamei*, e.g., 0.39 for survival time and 0.39 for survival status, between two different saline environments with high ammonia concentration. This suggests a strong G×E for ammonia tolerance across different salinity levels. Li, Luan [36] found that G×E for survival in different water temperatures was 0.29 ± 0.22 (*r*_g_) and suggested this trait could be controlled by different sets of loci in different temperatures for *P. vannamei*. Luan, Wang [37] reported variable estimates (low to high) of genotype-by-pond interaction (*r*_g_ = 0.001 ± 0.17 to 0.80 ± 0.06) for survival across different generations of *M. rosenbergii*. Gitterle, Salte [28] reported a low genotype-by-farm interaction for survival traits. A similar finding of high genetic correlation between replicated tanks for survival (WSSV resistance) was also reported by Gitterle, Salte [28]. Caballero-Zamora, Montaldo [38] reported moderate to high genetic correlation (ranging 0.49 to 0.93) for survival traits across different ponds, suggesting the general pond environment has a less pronounced impact on survival than other environmental conditions. Moss, Moss [35] examined the genetic correlation for survival of *P. vannamei* infected with different TSV isolates, which ranged from 0.35 to 0.99. This suggests that virulence can vary significantly among various strains of the same pathogenic species and can affect the G×E of survival of *P. vannamei*. Tan, Luan [41] examined the effect of density on the survival of *P. vannamei* and found low G×E (*r_g_* = 0.77 ± 0.09), suggesting the genetic component of variation in survival might be less affected by stocking density. Gitterle, Gjerde [31] found that survival traits displayed significant interaction of genotype-by-infection based on the infection protocol used (e.g., IO = individual oral vs. WB = water-borne infection) to infect the experimental *P. vannamei* with white spot syndrome virus (WSSV). These authors observed significant re-ranking of families when family breeding values were predicted based on the two infection protocols. There are several approaches for the inoculation of pathogens, including feeding of infected tissues, oral delivery, and intramuscular injection. This study provided insight about how these methods can be an important source of non-genetic variability for evaluating the genetic merit of animals [31]. Hayes, Gitterle [46] found higher G×E for survival between tanks (*r_g_* = 0.09) in *P. vannamei* and speculated that this phenomena occurred due to lack of genetic variation. Noble, Coman [48] found moderate G×E (*r_g_* = 0.35 to 0.58) for GAV-induced mortality across different groups of tanks challenged on different days. Moss, Moss [35] found a positive phenotypic correlation of 0.55 and 0.88 for mean family survival for TSV challenge test and growth in pond. Despite re-ranking, this positive correlation suggests that pathogen tolerance can be improved under commercial farm conditions by means of selective breeding. Coman, Crocos [51] examined the effect of temperature on growth and survival traits in *P. japonicus*, where the G×E accounted for 2.5 to 10.9% of the variability of the growth and survival traits, respectively. Coman, Crocos [52] examined the effect of density on the growth and survival of *P. japonicus* and found a small but significant G×E for growth but not for survival traits. The interaction accounted for 2.3% of the variation of growth. 

In shrimp breeding programs, the aim is to improve overall survival and resilience against multiple environmental disturbances. Survival is a complex trait which is characterized by its high variability in expression and is difficult to measure. We observed a substantial level of G×E and re-ranking of shrimp species for survival traits across studies (Figure 2). This level of re-ranking for survival traits can result from the presence of various mortality factors expressed in different environments, and there might be limited additive genetic variation for resilience against these factors. Hoffmann and Merilä [9] have proposed that changes in the genetic response of a trait due to unfavorable conditions can be caused by increases in environmental and phenotypic variance, and that this can cause a decrease in the relative importance of additive effects and variance because of limited expression of additive gene effects (i.e., lowered heritability). 

### 4.3. G×E in Other Economically-Important Traits

Aside from growth and survival, only a handful of studies have been conducted to examine the impact of G×E on other economically important traits such as feed efficiency, body composition and body color. The meta-correlation value (*r_g_*) of these traits was 0.48 ± 0.27. However, this is an overall composite estimate across traits and it is thus important to look into results from individual studies for specific details. Pérez-Rostro, Racotta [27] studied the impact of acute hypoxia on the expression of the body composition traits of *P. vannamei*. The authors reported that biochemical traits (e.g., body protein percent) showed variation depending on oxygen condition. However, the authors reported lack of significant G×E for lactate concentration in hypoxic or normoxic situations. Glencross, Tabrett [68] reported the existence of significant G×E on the impact of diet on growth in *P. monodon* and demonstrated that genetically selected shrimp can perform better if they are fed with an improved diet. A low-quality diet regime might have restricted the animal in expressing its full genetic potential. Dai, Kong [42] investigated the extent of G×E for feed efficiency traits in *P. vannamei* in different stocking density and conditions (e.g., individual vs. group reared). They found that moderate G×E was evident for the FER trait but was insignificant for growth expressed as ADG. Giang, Knibb [43] examined the G×E for body color in two environments (viz. two locations) and found it to be significant (*r_g_* = −0.40 to 0.16). This study signifies that rearing in different environments can improve the body color in shrimp. 

Overall, results from this meta-analysis identified the substantial presence of G×E in shrimp, which signifies the importance of implementing environment-specific breeding programs. For this purpose, “break-even correlation” or BEC can be used to decide whether running several environment-specific breeding programs will be cost effective or not. BEC is the point at which genetic gain is similar across different breeding plans. Under this scheme distinctive breeding strategies are recommended when *r_g_* across environments is lower than the BEC. For aquaculture species this BEC is assumed to be higher (e.g., ≥0.7) compared with livestock (e.g., 0.61 to 0.7) [69,70]. This is because, in aquaculture species, selection is generally carried out based on SIB testing combined with higher selection intensity [71]. Selection intensity in particular has a very large and proportional effect on BEC [69]. In our meta-analysis, *r_g_* values for growth, survival and other (feeding, body coloration and body composition) traits were 0.73 ± 0.06, 0.51 ± 0.09 and 0.62 ± 0.10, respectively. Therefore, higher genetic gain can be obtained for growth traits in different environments within a single breeding program in a common environment. However, environment-specific breeding programs will lead to higher genetic gain in shrimp species for survival and other traits. However, the breeder needs to consider the cost–benefit analysis in this kind of scenario [72]. 

### 4.4. Prospects to Overcome G×E in Breeding Programs 

G×E interaction has always been viewed as problematic in breeding programs under traditional selection breeding programs, which rely on pedigree information and performance testing; this becomes more complex when several environments need to be considered. However, genomic selection with reference populations reared in several environments can be far more effective [73]. Such multi-environment reference populations could be combined with high-throughput genomics and phenomics [74]. The use of large training datasets on genotypes and phenotypes can be utilized to select animals for improved resilience across environments. The multi-environment reference population can facilitate the prediction of accurate genomic enabled breeding values (GEBV) for the genotyped selection candidates. This is because genotyping and phenotyping of large number of selection candidates increases the possibility of creating specialized lines by genomic selection methods, coupled with the BEC usually being higher with genomic selection than with a conventional selection breeding approach [75]. By utilizing genetic variance among populations, G×E can play a significant positive role for increasing resilience. Mulder [75] compared the accuracy of EBVs in an extreme environment by using a reaction norm (RN) model, which considers G×E interactions, and a conventional model, which ignores them. The study found that the RN model yielded higher EBV accuracy. Furthermore, the author investigated the effectiveness of incorporating G×E information in genomic selection using large reference populations. The results demonstrate that the RN model was capable of reducing environmental sensitivity by selecting resilient animals. This method of GS outperformed traditional breeding methods by 9 to 140% across environments [75]. The author evaluated the advantage of genomic selection in fish in the presence of G×E, where the production performance of the reference population was measured in commercial farms, and found that the selection accuracy greatly improved under such a scheme. Chu, Alemu [76] evaluated the impact of level of G×E on genetic gain in the genomic breeding schemes for broiler chicken and found that when G×E is strong (*r_g_* = 0.5 to 0.7), genomic selection of birds reared in a commercial production environment yielded higher genetic gain than when production data from the bio-secure environment was used. From these studies it is becoming clear that G×E can be managed successfully by using genomic information whilst increasing the accuracy of EBV estimation, provided that the reference population is large. Once the models are trained using data from diverse environments, GS in aquaculture shrimp species can be performed in the centralized nucleus facilities. This approach eliminates the need for progeny testing across different environments.

In the face of changing global climatic conditions, the incorporation of G×E information in future breeding programs will be crucial [77]. Aquaculture seed stocks adapted to current environmental conditions may not perform optimally in future changing conditions and climate change may cause a sudden outbreak of existing and novel pathogens. This becomes even more important when popular seed stocks are being used across the globe. To overcome these challenges, the application of selective breeding to develop resilient animals will be of great importance [78]. 

Disease in shrimp farming is a major constraint to obtain profitable yields. Most shrimp pathogens are transmitted vertically, and disease is often the result of a massive viral amplification generally triggered by various environmental and physiological stresses. Based on detailed environmental data, the likelihood of pathogen attack can be predicted both in the short and long term [79]. Such predictions can potentially be carried out for the coming year or season based on currently available weather forecast data in combination with historical data on diseases, pathogens and climatic factors [80]. The ability to predict phenotypes in a specific environment is important to manage disease and pests in a sustainable and dynamic way. Although no such studies have been conducted in shrimp, studies on wheat have shown that specific disease outbreaks can be successfully predicted for different environments. For example, wheat rust Ug99 disease dynamics/epidemics have been predicted in east Africa by integrating the current status and distribution of the rust, prevailing winds, climatic factors for rust survival and sporulation, wheat production zones, historical migration patterns of rust races, and responses to the existing cultivars to the rusts [81]. Similar approaches can be taken to develop disease-resistant shrimp. To attain this, detailed environmental data, along with genotype and phenotype data, need to be considered in the breeding programs [82]. This detailed environmental data should include multiple environmental trials, geographic and historical weather information, measurement of habitat characteristics (e.g., pond soil and water properties) and the evaluation of companion organisms (e.g., susceptible pathogens and microbes) at an ecosystem level. So far, variation in environmental factors has not been properly integrated into shrimp breeding programs. This is largely due to the fact that environmental factors have been considered at a gross level, e.g., only major environmental parameters are considered such as location and station and is generally treated as a ‘black box’ effect that interacts with the genotype to affect phenotype without considering individual components. Moreover, environmental parameters are dynamic throughout the animal grow-out periods. To circumvent these limitations, precise dissection of complex environmental factors for both target environments and for specific genotypes needs to be carried out for sustainable management, control and optimization of environmental factors for enhanced genetic improvement program [82,83,84]. Therefore, genomic selection and use of ‘big data’ can be successfully utilized to exploit G×E as a ‘game changer’ tool of genetic improvement across environments.

Throughout our review it is apparent that there is a lack of strategies for assessing a large number of individual family pedigrees. In many cases, a mass selection method is applied in shrimp breeding, where animals with superior phenotypic values for target traits are chosen as the breeders for the next generation. However, if individuals are chosen in the absence of particular environmental factors (e.g., disease factors) that are highly correlated, then, over generations, the performance of the animal for the selected traits might decrease. Alternatively, a family-based selection scheme considers multiple-trait expression in different environments, which provides a powerful tool with which to improve multiple traits simultaneously. Therefore, to better manage the G×E issue in shrimp breeding, family-based selection or a genomic selection scheme should be preferred. 

## 5. Conclusions and Recommendation

Most of the studies have focused on only a single major shrimp species used in commercial aquaculture (e.g., *P. vannamei*), with the main traits of interest being growth and survival. However, many other economically important species and traits (e.g., feed efficiency, nutrition, body composition and body color) also need to be considered for G×E studies. In addition, our review revealed that there is a lack of standard documentation for G×E effects across studies. For proper utilization of G×E information in breeding programs and to ensure reproducibility, minimum standard information should be provided from each study (e.g., family information, population size, and a standardized definition of environmental parameters etc.). 

This review underscores the potential of genomic selection in addressing G×E interactions for shrimp breeding programs. Conventional practices of rearing shrimp families in individual tanks or ponds can lead to confounding of genetic and environmental effects, whereas communal family rearing with marker-based pedigree estimation mitigates these effects and improves the accuracy of genomic breeding values. To optimize breeding programs, it is crucial to ensure that disease challenge test conditions closely resemble grow-out environments, and that environmental information is incorporated into the selection index. Controlling G×E effects can be achieved by maintaining separate genetic lines or assessing sibling performance in different environments, combining evaluation data to maintain genetic diversity.

Traditionally, separate tanks/ponds are used for producing shrimp families for maintaining genetic lines and the evaluation of traits. This facilitates easy tracing of families and is convenient for stocking equal numbers of animals/individuals per family. This separate rearing environment can introduce a significant confounding of family traits with tank-specific environment effects. In contrast, as demonstrated by Jerry, Preston [53], communal family rearing has been proposed to eliminate such effects by means of marker-based pedigree estimation. This can ultimately eliminate the potential non-genetic effects (e.g., common environment effect due to rearing system) and increase the reliability of measures of genetic value [85]. 

The measurement of disease resistance through a disease challenge test is only effective for selection breeding programs where the challenge conditions are very similar to the grow-out conditions. Therefore, it is critically important to optimize the grow-out environment to be as similar as possible to the selection environment. It can be done by incorporating production environment information in the selection index. To limit re-ranking, maintaining separate genetic lines for different environments is essential to secure long-term response to the selection and maintenance of genetic diversity. Another approach to control G×E effects would be to test sibling production performance in different production environments and to use combined genetic evaluation of the production data from both environments. 

## Figures and Tables

**Table 1:** Details of the G×E studies in commercially farmed shrimp species.

Species	Publication Year	Country	Traits	Environment	References
*P. vannamei*	2002	USA	Growth	Pond vs. tank	Argue, Arce [24]
*P. vannamei*	2003	Mexico	Growth	Tank A vs. B	Pérez-Rostro and Ibarra [25]
*P. vannamei*	2003	Mexico	Growth	Environment 1 vs. Environment 2	Pérez-Rostro and Ibarra [26]
*P. vannamei*	2004	Mexico	Biochemical compounds (e.g., protein, lactate, carbohydrate)	Normoxic vs. hypoxic condition	Pérez-Rostro, Racotta [27]
*P. vannamei*	2005	Colombia	Growth, survival	Pathogenic stress	Gitterle, Salte [28]
*P. vannamei*	2005	Colombia	Growth, survival	Pond vs. tank	Gitterle, Rye [29]
*P. vannamei*	2006	Colombia	Survival	Pathogenic stress	Gitterle, Ødegård [30]
*P. vannamei*	2006	Colombia	Survival	Pathogenic stress	Gitterle, Gjerde [31]
*P. vannamei*	2007	Mexico	Growth	Stocking density	Castillo-Juárez, Casares [32]
*P. vannamei*	2008	Mexico	Growth	Stocking density	Ibarra and Famula [33]
*P. vannamei*	2009	Mexico	Growth	Environment 1 vs. Environment 2	Campos-Montes, Montaldo [34]
*P. vannamei*	2013	USA	Survival	Pathogen A vs. B	Moss, Moss [35]
*P. vannamei*	2015	China	Growth, survival	Temperature	Li, Luan [36]
*P. vannamei*	2015	China	Growth	Stocking density	Luan, Wang [37]
*P. vannamei*	2015	Mexico	Growth, survival	Ponds	Caballero-Zamora, Montaldo [38]
*P. vannamei*	2016	China	Growth	Farms	Sui, Luan [39]
*P. vannamei*	2017	China	Growth, survival	Salinity levels	Lu, Luan [40]
*P. vannamei*	2017	China	Growth, survival	Stocking density	Tan, Luan [41]
*P. vannamei*	2019	China	Feed efficiency	Stocking density	Dai, Kong [42]
*P. vannamei*	2019	Vietnam	Growth, body color	Environments	Giang, Knibb [43]
*P. vannamei*	2020	Vietnam	Growth	Pond vs. tank	Nguyen, Ninh [44]
*P. vannamei*	2021	Mexico	Growth, survival	Pathogenic stress	Cala-Moreno, Campos-Montes [45]
*P. monodon*	2010	India	Survival	Tanks	Hayes, Gitterle [46]
*P. monodon*	2011	India	Growth, survival	Ponds	Krishna, Gopikrishna [47]
*P. monodon*	2020	Australia	Growth, survival	Pathogenic stress	Noble, Coman [48]
*P. monodon*	2020	Vietnam	Growth, survival	Recirculating system vs. ponds	Van Sang, Luan [7]
*P. monodon*	2021	China	Growth, survival	Diets	Jiang, Mo [49]
*P. monodon*	2022	Australia	Growth	Ponds	Hasan, Thomson [50]
*P. japonicus*	2002	Australia	Growth, survival	Temperature	Coman, Crocos [51]
*P. japonicus*	2004	Australia	Growth, survival	Stocking density	Coman, Crocos [52]
*P. japonicus*	2006	Australia	Growth	Ponds	Jerry, Preston [53]
*M. rosenbergii*	2013	China	Growth, survival	Ponds	Luan, Wang [37]

**Table 2:** Definition of groups of traits and environment.

Trait	Definition
Growth	Body weight, growth rate, average daily gain (ADG), body length.
Survival	Overall (end of the culture period) or in a challenge test.
Other traits	Body composition, protein, lipid, carbohydrate content, body color, feed efficiency ratio (FER).
**Environment**	
Habitat	Pond vs. pond (location), pond vs. tank or cage.
Stress	Salinity, ammonia concentration, temperature, density (high vs. low), pathogens.

**Table 3:** Meta-estimates of genetic correlation across trait groups (**a**) and environments (**b**). “*” indicates that genetic correlation is significantly different between family groups (*p* < 0.05).

(**a**)		
Trait group	Meta Genetic Correlation	Group differences
Growth	0.72 ± 0.05	*
Survival	0.58 ± 0.07	*
Other traits	0.48 ± 0.27	*
(**b**)		
Environments	Meta Genetic Correlation	Group differences
Habitat type	0.65 ± 0.07	NS
Stress	0.67 ± 0.08	NS

Group differences: * = significant; NS = non-significant.

**Table 4:** Variance explained by G×E from ANOVA-based studies.

Traits	Environment	Source of Variation (or G×E)	Variation Explained by G×E (%)	Reference
Survival	Density	Density∗Family	0.3	Coman, Crocos [52]
Growth	Density	Density∗Family	1.95	Coman, Crocos [52]
Growth	Hypoxic condition	Habitat∗Family	7.78	Pérez-Rostro, Racotta [27]
Growth	Hypoxic condition	Habitat∗Family	9.23	Pérez-Rostro, Racotta [27]
Growth	Hypoxic condition	Habitat∗Family	16.65	Pérez-Rostro, Racotta [27]
Growth	Hypoxic condition	Habitat∗Family	6.06	Pérez-Rostro, Racotta [27]
Growth	Hypoxic condition	Habitat∗Family	4.98	Pérez-Rostro, Racotta [27]
Growth	Hypoxic condition	Habitat∗Family	7.03	Pérez-Rostro, Racotta [27]
Growth	Hypoxic condition	Habitat∗Family	4.7	Pérez-Rostro, Racotta [27]
Growth	Temperature	Temperature∗Family	4.68	Coman, Crocos [51]
Growth	Temperature	Temperature∗Family	3.74	Coman, Crocos [51]
Survival	Temperature	Temperature∗Family	11.63	Coman, Crocos [51]
Survival	Temperature	Temperature∗Family	9.46	Coman, Crocos [51]
Growth	Temperature	Temperature∗Family	6.93	Coman, Crocos [51]
Growth	Temperature	Temperature∗Family	7.96	Coman, Crocos [51]
Growth	Habitat	Habitat∗Family	1.8	Argue, Arce [24]
